# RNA modifications in cancer stem cells: molecular mechanisms and targeted therapeutic strategies

**DOI:** 10.3389/fcell.2026.1818554

**Published:** 2026-03-25

**Authors:** Jiankun Zhang, Yixiao Yuan, Chongxin Li, Xiulin Jiang, Weiqiang Song

**Affiliations:** 1 Department of Urology, Hebei Petro China Central Hospital, Langfang, China; 2 The First Affiliated Hospital of Chongqing Medical University, Chongqing, China; 3 Qujing Central Hospital of Yunnan Province, Qujing, China; 4 Department of Systems Biology, Beckman Research Institute of City of Hope, Monrovia, CA, United States

**Keywords:** AC4C, cancer stem cells, m1A, m5C, M6A, m7G, RNA modifications, self-renewal

## Abstract

Cancer stem cells (CSCs) are a key subpopulation within tumors, characterized by their self-renewal and differentiation potential, and they drive tumor initiation, progression, metastasis, and recurrence. Recent epitranscriptomic studies have revealed that RNA modifications, including m^6^A, m^5^C, ac^4^C, m^7^G, and m^1^A, play critical roles in maintaining CSC stemness, determining cell fate, reprogramming metabolism, and promoting therapy resistance. This review systematically summarizes the functions of different RNA modifications and their associated enzymes in CSCs. We also discuss how RNA modifications regulate core CSC signaling pathways, such as Wnt/β-catenin, Notch, Hedgehog, PI3K–AKT–mTOR, JAK/STAT, Hippo/YAP, and TGF-β/SMAD, and we highlight strategies targeting RNA modifications for CSC intervention along with their potential challenges. These findings suggest that RNA modifications and their regulators represent promising therapeutic targets in CSCs, providing a rationale for developing highly selective or combination treatment strategies.

## 1 Introduction

CSCs are a subpopulation of tumor cells with stem cell-like properties, playing critical roles in tumor initiation, progression, and recurrence (Toh et al., 2017). The key features of CSCs include long-term self-renewal, the ability to generate heterogeneous cell populations within tumors through differentiation plasticity, and intrinsic resistance to conventional chemotherapy and radiotherapy (Amiri-Farsani et al., 2024). These properties allow CSCs to survive treatment, form minimal residual disease, and drive tumor relapse and metastasis. Recent studies have shown that CSCs are not a fixed cell population but exhibit dynamic plasticity, and their stemness can be finely regulated by multiple molecular mechanisms (Amiri-Farsani et al., 2024).

Building on epigenetic and transcriptomic research, epitranscriptomics has emerged as a field highlighting reversible RNA modifications as critical regulators of gene expression (Barbieri and Kouzarides, 2020). Common RNA modifications include N6-methyladenosine (m^6^A), 5-methylcytidine (m^5^C), N4-acetylcytidine (ac^4^C), 7-methylguanosine (m^7^G), and 1-methyladenosine (m^1^A) (Roundtree et al., 2017). These modifications can regulate RNA stability, splicing, translation efficiency, and non-coding RNA function, enabling rapid, reversible, and precise control of gene expression (Roundtree et al., 2017). This layer of regulation provides new molecular mechanisms for the dynamic maintenance and fate determination of CSCs.

Therefore, this review aims to summarize recent advances, focusing on how RNA modifications regulate CSC stemness, self-renewal, metabolic reprogramming, cell fate decisions, and therapy resistance, providing insights into CSC biology and potential strategies for targeted intervention.

## 2 Overview of RNA modifications

### 2.1 m^6^A

m^6^A is the most abundant methylation modification in eukaryotic mRNA, occurring on adenosine (A) and typically enriched in the 3′untranslated region (3′UTR), near stop codons, and in long exons (Jiang et al., 2021). Its deposition is catalyzed by the “Writer” complex, including METTL3, METTL14, WTAP, METTL16, and RBM15. Demethylases (“Erasers”) include FTO and ALKBH5, while “Readers” such as YTHDF1-3, YTHDC1-2, and IGF2BP1-3 recognize m^6^A and regulate RNA stability, splicing, and translation efficiency, thereby controlling gene expression (Jiang et al., 2021).

### 2.2 m^5^C

m^5^C occurs on cytosine (C) and is present in mRNA, tRNA, and rRNA, contributing to RNA stability and nuclear export (Chen et al., 2025). Key writers include NSUN1-7 and DNMT2; erasers include TET1-3, ALKBH1, and FTO; readers include YBX1 (Li et al., 2025a), YBX2, ALYREF (Li et al., 2025b), SRSF2, and YTHDF2. These proteins bind m^5^C-modified RNA to regulate its stability and transport (Chen et al., 2025).

### 2.3 ac^4^C

ac^4^C is another cytidine modification, mainly catalyzed by NAT10. This modification enhances RNA translation efficiency and stability (Li et al., 2025c), positively regulating protein synthesis. In CSCs, NAT10-mediated ac^4^C modification may maintain self-renewal by promoting the expression of key stemness genes (Wang et al., 2025a).

### 2.4 m^7^G

m^7^G modification occurs on guanosine (G) and is widely found in the 5′cap of mRNA and in tRNA (Zhao et al., 2023). Writers include RNMT/RAM, METTL1/WDR4, and WBSCR22/TRMT112. m^7^G regulates RNA stability and translation initiation, representing an important post-transcriptional modification (Orellana et al., 2021).

### 2.5 m^1^A

m^1^A occurs on adenosine (A) and is mainly present in tRNA, rRNA, and a small fraction of mRNA (Oerum et al., 2017). Writers include TRMT6/TRMT61A and TRMT10C, while the eraser is ALKBH3. m^1^A can alter RNA secondary structure, regulate translation efficiency, and influence RNA stability (Wang et al., 2024a).

## 3 Functions and mechanisms of RNA modifications in CSC

RNA modifications play critical roles in regulating CSC properties, including self-renewal, proliferation, metastasis, and therapy resistance. Multiple types of RNA modifications, such as m^6^A, m^5^C, ac^4^C, m^1^A, and m^7^G, are catalyzed or recognized by specific enzymes and RNA-binding proteins, which control the stability, translation, or nuclear export of key stemness-associated transcripts. For example, m^6^A writers (METTL3, METTL14, METTL16, WTAP, RBM15), erasers (ALKBH5, FTO), and readers (YTHDF1/2, IGF2BP family, YTHDC1) modulate CSC traits across diverse cancers. Similarly, m^5^C writers (NSUN2, TET family) and readers (YBX1, YBX2, hnRNPA2B1) maintain CSC stemness and therapy resistance. The ac^4^C writer NAT10, m^1^A methyltransferase TRMT6/TRMT61A, and m^7^G writer METTL1 further contribute to CSC self-renewal, metabolic regulation, and tumor progression. In Table 1, we summarizes the key RNA-modifying enzymes, their target genes, the type of modification and effect, the CSC type, and the associated CSC phenotypes, highlighting the diverse mechanisms by which RNA epigenetic regulation sustains cancer stemness and malignancy.

**TABLE 1:** Key RNA modifications and their functional roles in cancer stem cells (CSCs).

Regulator	Target	Effect	CSC type	Function
METTL3	NFE2L3	m^6^A-mediated stabilization	Lung adenocarcinoma (OV6^+^ CSCs)	Self-renewal, immune suppression, chemoresistance
METTL3	SOCS3	m^6^A-mediated regulation	Liver CSCs	Self-renewal, tumorigenicity
METTL3	PTGER2	m^6^A-mediated upregulation	Ovarian CSCs	Self-renewal, EMT, DNA repair, metastasis, chemoresistance
METTL14	ATF4/PHGDH/PSAT1	m^6^A-mediated stabilization	AML LSCs/LICs	Metabolic reprogramming, proliferation, self-renewal
METTL14	YAP1	Reduced m^6^A, decreased degradation	TNBC CSCs	Self-renewal, Hippo pathway activation
METTL14	SCD1/ATF5	m^6^A-mediated degradation	CRC/GC CSCs	Suppression of Wnt/β-catenin, decreased CSC stemness, proliferation, migration
METTL14	PKM2	m^6^A-mediated nuclear translocation via YTHDF1	NSCLC CSCs	Self-renewal, glycolysis, ferroptosis resistance
METTL16	eIF3a	m^6^A-mediated translation regulation	HCC CSCs	Self-renewal, tumorigenicity
METTL16	BCAT1	m^6^A-mediated translation, BCAA metabolism	AML LSCs/LICs	Self-renewal, proliferation
WTAP	LOXL2	m^6^A-mediated stabilization via IGF2BP2	GBM CSCs	EMT, M2 macrophage polarization, immune evasion
WTAP	GBE1	m^6^A-mediated stabilization via IGF2BP3	Pancreatic CSCs	CSC-like traits, proliferation, tumor progression
WTAP	EGR1	m^6^A-mediated stability via IGF2BP3	Endometrial CSCs	Self-renewal, cisplatin resistance
RBM15	HEIH	m^6^A-mediated stabilization	Cervical CSCs	Self-renewal, proliferation, migration
RBM15	TPM1	m^6^A-mediated regulation	Prostate CSCs/CRPC	Stemness, apoptosis regulation, chemoresistance
ALKBH5	SOX4	m^6^A demethylation	Liver CSCs	Self-renewal, SHH pathway activation, M2 macrophage polarization
ALKBH5	FAM84A	m^6^A demethylation	Colorectal CSCs	Stemness, chemoresistance via β-catenin stabilization
ALKBH5	TACC3	m^6^A demethylation	AML LSCs/LICs	Self-renewal, tumorigenicity
FTO	LILRB4	m^6^A/m^6^Am demethylation	AML LSCs/LICs	Immune evasion inhibition, self-renewal
FTO	EMT genes	m^6^A/m^6^Am demethylation	Pancreatic CSCs	Stemness, proliferation, migration, invasion
FTO	PDE1C/PDE4B	m^6^Am demethylation	Ovarian CSCs	Suppression of stemness, tumorigenicity
YTHDF1	NOTCH1	m^6^A-mediated stabilization & translation	HCC/CRC CSCs	Self-renewal, chemoresistance
YTHDF1	SIAH2	Activation of Hippo pathway	TNBC CSCs	Stemness, proliferation, chemoresistance
YTHDF1	TCF7L2/TCF4	m^6^A-mediated translation	ISC/Wnt-driven tumors	Stemness, tumorigenesis
YTHDF2	ONECUT2	m^6^A-mediated degradation	Gastric CSCs	Suppression of stemness, drug resistance
YTHDF2	SOX2	m^6^A-mediated stabilization via LINC00511	Cholangiocarcinoma CSCs	Stemness, malignant phenotype
YTHDF2	GLI2	m^6^A-mediated regulation	Cervical CSCs	Inhibition of stemness, apoptosis promotion
YTHDF2	OCT4	m^6^A-mediated 5′-UTR translation	HCC CSCs	Stemness, metastasis
IGF2BP1/2	CDC45/circ_0000745/EMX2OS	m^6^A-mediated stabilization	HCC/OC/Wilms' tumor CSCs	Stemness, proliferation, migration, invasion
YTHDC1	circFNDC3B	Nuclear-cytoplasmic transport	CRC CSCs	Inhibition of stemness and metastasis
YTHDC1	MCM4	m^6^A-mediated regulation	AML LSCs	Self-renewal, proliferation
YTHDC1	General m^6^A targets	m^6^A-mediated recognition	HNSCC CSCs	Enhanced stemness, poor clinical outcome
NSUN2	PGK1	m^5^C-mediated stabilization via YBX1	Gastric CSCs	Stemness, proliferation, invasion, glycolysis
TET1	OCT4, CCNB1/CDK1	m^5^C/hm^5^C-mediated regulation	Breast CSCs	Stemness, cell cycle control
TET1	CCNY/CDK16	m^5^C/hm^5^C-mediated regulation	Renal CSCs	Proliferation, cell division
TET2 loss	TSPAN13	Increased m^5^C, YBX1 recognition	AML LSCs	Homing, proliferation, self-renewal
YBX1	NANOG	Transcription activation	NSCLC CSCs	Stemness, invasion, tumor formation
YBX1	SOX2 via lncTCFL5-2	Protein stabilization	RCC CSCs	Stemness, drug resistance (Sunitinib)
YBX1	SOX9	Transcription/translation activation	ICC CSCs	Stemness, cisplatin resistance via AKT/β-catenin
YBX1	MUC1	Promoter activation	Lung adenocarcinoma CSCs	Stemness, migration
circRABL2B	MUC5AC via YBX1	Inhibition of target expression	Lung CSCs	Suppression of CSC traits, increased drug sensitivity
YBX2	CT45	Transcriptional upregulation	Endometrial CSCs	Self-renewal, chemoresistance (paclitaxel)
hnRNPA2B1	m^6^A-marked	Nuclear export via ALY/REF-NXF1	Breast CSCs (BCSCs)	Stemness maintenance, chemoresistance
NAT10	SLC1A4, HOXA9, MENIN	ac^4^C-mediated translation enhancement	AML CSCs	Self-renewal, tumorigenesis
NAT10	PGAM1, CAPRIN1	ac^4^C-mediated mRNA stabilization	Colorectal/Ovarian CSCs	Sphere formation, migration, invasion, chemoresistance
NAT10	JARID2	ac^4^C-mediated stabilization	GBM CSCs	Stemness, malignant progression
NAT10	CEBPA	ac^4^C-mediated stabilization	NSCLC CSCs	Stemness, proliferation, migration, tumor growth

### 3.1 m^6^A in cancer stem cells

#### 3.1.1 Functions and mechanisms of m^6^A writers in CSC

METTL3, a core m^6^A methyltransferase, plays a central role in various CSCs. It maintains CSC self-renewal, proliferation, and therapy resistance by stabilizing or enhancing the translation of key target mRNAs via m^6^A modification. METTL3 inhibitors (STM2457) or autophagy inhibitors (HCQ) effectively suppress BRAF V600E-driven glioblastoma (GBM) progression (Xie et al., 2025). In ovarian cancer, METTL3-mediated m^6^A modification upregulates PTGER2, enhancing CSC self-renewal, epithelial–mesenchymal transition (EMT), DNA damage repair, metastasis, and cisplatin resistance (Lin and Xu, 2023). METTL14, another core m^6^A methyltransferase, similarly regulates CSC self-renewal, stemness, and therapy resistance. In acute myeloid leukemia (AML), METTL14 collaborates with the m^6^A reader IGF2BP3 to stabilize serine synthesis pathway (SSP) genes (ATF4, PHGDH, PSAT1), supporting LSC metabolic demands and proliferation, revealing metabolic vulnerabilities (Huang et al., 2025). In NSCLC, SETD5-mediated H3K36me3 promotes METTL14-dependent m^6^A modification, recruits YTHDF1, and regulates PKM2 nuclear translocation and SOX9/GPX4 signaling, enhancing CSC stemness, glycolysis, and ferroptosis resistance, facilitating metastasis (Liu et al., 2025a). METTL16, a newly identified m^6^A methyltransferase, is highly expressed in hepatocellular carcinoma (HCC) CSCs. Its depletion reduces CSC frequency and tumor growth with minimal effects on normal hepatocytes. METTL16 regulates rRNA maturation and mRNA translation, with eIF3a identified as a functional target, providing a basis for potential inhibitors (Su et al., 2022). In AML, METTL16 is essential for LSC/LIC survival and self-renewal, promoting expression of BCAT1/BCAT2 via m^6^A and reprogramming branched-chain amino acid metabolism to maintain CSC stemness and proliferation (Han et al., 2023).

WTAP, a critical component of the m^6^A methyltransferase complex, is stabilized by OGT-mediated O-GlcNAcylation and USP7-mediated deubiquitination (Qiu et al., 2025). In GBM, WTAP m^6^A-modifies LOXL2 mRNA, with IGF2BP2 maintaining its stability, promoting mesenchymal transition and M2 macrophage polarization, enabling immune evasion (Qiu et al., 2025). In pancreatic cancer, WTAP stabilizes GBE1 mRNA via IGF2BP3, promoting CSC traits and tumor progression (Jin et al., 2024). In endometrial cancer, WTAP downregulation reduces m^6^A modification, destabilizes EGR1 mRNA, downregulates PTEN, and enhances CSC stemness, self-renewal, and cisplatin resistance, indicating the WTAP/EGR1/PTEN axis as a therapeutic target (Wang et al., 2024b). RBM15, another m^6^A complex component, stabilizes oncogenic lncRNAs such as HEIH in cervical cancer, promoting CSC self-renewal via miR-802/EGFR regulation (Quan et al., 2024). In prostate cancer, RBM15 modifies TPM1 mRNA through m^6^A methylation to regulate its stability. The pseudogene-derived lncRNA AZGP1P2 enhances this effect by stabilizing RBM15 protein. Mechanistically, AZGP1P2 interacts with RBM15 and the E1 ubiquitin-activating enzyme UBA1, thereby interfering with UBA1-mediated ubiquitination and subsequent degradation of RBM15, which ultimately promotes RBM15-dependent m^6^A modification and TPM1 mRNA decay (Quan et al., 2024).

#### 3.1.2 Functions and mechanisms of m^6^A demethylases in CSCs

m^6^A demethylases, particularly ALKBH5 and FTO, play critical roles in regulating CSC maintenance, metabolic adaptation, and therapy resistance across diverse tumor types. Among them, ALKBH5 has been widely reported to sustain CSC stemness through multiple regulatory mechanisms. For example, USP14 deubiquitinates and stabilizes ALKBH5, thereby enhancing glioma stem cell (GSC) stemness, self-renewal, and radioresistance via the MST4–USP14–ALKBH5 signaling axis (Zhou et al., 2025a). Furthermore, ALKBH5 regulates α-KG/L-2-HG metabolism to maintain mitochondrial energy supply, thereby promoting the self-renewal and clonogenic capacity of hematopoietic stem/progenitor cells and AML stem cells (Gao et al., 2023). In glioma, EGFR activates SRC signaling to retain ALKBH5 in the nucleus, maintaining m^6^A demethylation, inhibiting ferroptosis, and enhancing chemoresistance in GSCs (Lv et al., 2023). Similarly, ALKBH5 demethylates SOX4 mRNA, activating the SHH signaling pathway and promoting both liver cancer stem cell stemness and M2 macrophage polarization (Yang et al., 2023). In addition, elevated ALKBH5 expression regulates downstream targets such as TACC3, maintaining the self-renewal and tumorigenicity of AML stem/initiating cells (LSC/LIC) (Shen et al., 2020). Upstream regulation of ALKBH5 is also observed in leukemia, where KDM4C increases ALKBH5 expression and stabilizes AXL mRNA, thereby sustaining AML stem cell function (Wang et al., 2020). In contrast to ALKBH5, the m^6^A demethylase FTO exhibits context-dependent roles in CSC regulation. In AML, FTO inhibits m^6^A/m^6^Am modifications and suppresses immune evasion by reducing LILRB4 expression, thereby enhancing the self-renewal capacity of AML stem/initiating cells (Wang et al., 2020). Furthermore, high FTO expression promotes pancreatic cancer CSC stemness by enhancing EMT, tumor sphere formation, and CSC marker expression, ultimately facilitating proliferation, migration, and invasion. However, in colorectal cancer, reduced FTO expression increases CSC stemness, tumorigenicity, and chemoresistance through dysregulated m^6^Am modification, a phenotype that can be reversed by inhibition of PCIF1/CAPAM (Li et al., 2022). Similarly, low FTO expression has been observed in ovarian CSCs, where FTO overexpression reduces the stability of PDE1C and PDE4B mRNAs and activates cAMP signaling, thereby suppressing CSC stemness and tumorigenesis (Huang et al., 2020a). Collectively, these findings highlight that although both ALKBH5 and FTO regulate CSC properties through m^6^A or m^6^Am demethylation, their functional effects are highly context-dependent and can vary across tumor types, reflecting the complex epitranscriptomic regulation underlying CSC maintenance and therapeutic resistance.

#### 3.1.3 Functions and mechanisms of m^6^A readers in CSCs

m^6^A reader proteins recognize m^6^A-modified transcripts and regulate their stability, translation, and localization, thereby playing crucial roles in maintaining CSC properties and tumor progression. Among them, members of the YTH domain family, particularly YTHDF2 and YTHDC1, have been widely implicated in the regulation of CSC stemness in different tumor contexts. In gastric cancer, YTHDF2 promotes the degradation of ONECUT2 mRNA in an m^6^A-dependent manner, thereby suppressing CSC stemness and drug resistance (Fan et al., 2025). In contrast, in cholangiocarcinoma, YTHDF2 interacts with lncRNA LINC00511 to stabilize SOX2 mRNA through m^6^A recognition, thereby maintaining CSC stemness and promoting malignant phenotypes (Guan et al., 2024). These findings highlight the context-dependent role of YTHDF2 in regulating the stability of key stemness-related transcripts. Another YTH family member, YTHDC1, primarily functions in the nucleus and is also critical for CSC maintenance and tumor progression. In head and neck squamous cell carcinoma (HNSCC), high YTHDC1 expression is associated with enhanced stemness and poor clinical outcomes, contributing to the maintenance of malignant epithelial cell stemness. In colorectal cancer (CRC), YTHDC1 promotes the cytoplasmic export of m^6^A-modified circFNDC3B and suppresses stemness and metastasis through the circFNDC3B/FXR2/RNF41/ASB6 regulatory axis (Zeng et al., 2022). In acute myeloid leukemia (AML), YTHDC1 maintains LSC self-renewal and proliferation by regulating the DNA replication factor MCM4 (Sheng et al., 2021). Moreover, the small-molecule inhibitor YL-5092 disrupts YTHDC1 binding to m^6^A-modified substrates, reduces mRNA stability, and selectively depletes LSCs without affecting normal hematopoietic stem cells, highlighting YTHDC1 as a promising therapeutic target for CSC maintenance and therapy resistance (Zhang et al., 2026). Collectively, these studies demonstrate that YTH family readers regulate CSC phenotypes by modulating the fate of m^6^A-modified transcripts involved in stemness and tumor progression.

Another important class of m^6^A readers is the insulin-like growth factor 2 mRNA-binding protein (IGF2BP) family, which primarily enhances the stability and translation of m^6^A-modified mRNAs, thereby promoting CSC traits and tumor progression. In Wilms’ tumor, the lncRNA EMX2OS binds IGF2BP1 and suppresses tumor cell stemness, migration, invasion, and EMT, functioning as a tumor suppressor by disrupting IGF2BP1-mediated RNA regulation (Zhang et al., 2023). In HCC, IGF2BP2 interacts with CDC45 mRNA in an m^6^A-dependent manner, promoting CSC stemness, proliferation, migration, invasion, EMT, and glycolysis while inhibiting apoptosis (Wu et al., 2023). Overall, these findings suggest that IGF2BP family proteins contribute to the maintenance of CSC characteristics mainly by stabilizing oncogenic m^6^A-modified transcripts, further highlighting the diverse regulatory roles of m^6^A readers in CSC biology across different tumor types.

### 3.2 Functions and mechanisms of m^5^C in CSCs

The ten–eleven translocation (TET) family of dioxygenases plays important roles in CSC maintenance and tumor progression (Joshi et al., 2022). Traditionally, TET proteins—including TET1, TET2, and TET3—are recognized as DNA demethylases that catalyze the oxidation of 5-methylcytosine (5mC) to 5-hydroxymethylcytosine (5hmC), thereby regulating DNA methylation dynamics and gene transcription (Joshi et al., 2022). However, emerging evidence indicates that TET enzymes can also function in RNA epigenetic regulation, acting as demethylases for RNA m^5^C and thereby influencing mRNA stability and translation (Li et al., 2023). Functionally, TET family members regulate CSC stemness and tumor progression through multiple mechanisms. In breast cancer, TET1 upregulates OCT4 expression and regulates the CCNB1/CDK1 axis, thereby maintaining CSC stemness and controlling cell-cycle progression (Bartoccetti et al., 2020). Consistently, in renal cancer HuR-positive CSCs, the flavonoid fisetin suppresses CSC proliferation and division by inhibiting TET1 activity and reducing local 5hmC modification, which in turn downregulates CCNY/CDK16 expression (Si et al., 2019). Moreover, TET2 plays a crucial role in LSC biology. In AML, loss of TET2 increases m^5^C modification of TSPAN13 mRNA, which is recognized by the m^5^C reader YBX1, thereby enhancing LSC homing to the bone marrow as well as their proliferation and self-renewal. In addition, accumulated m^5^C triggers MBD6-mediated deubiquitination of H2AK119ub, resulting in chromatin opening and enhanced transcription, ultimately making TET2-deficient AML cells dependent on this regulatory pathway (Li et al., 2023). Overall, TET enzymes maintain CSC stemness, proliferation, and therapy resistance via m^5^C/m5hmC modification and key target gene stability, offering potential strategies for targeting CSCs and TET-deficient tumors.

YBX1 is a well-characterized m^5^C RNA modification reader that binds m^5^C-modified transcripts and regulates their stability, translation, and transcriptional programs, thereby controlling key targets involved in CSC maintenance, tumor progression, and therapy resistance (Zhao et al., 2025a; Meng et al., 2024). In RCC, low AR induces lncTCFL5-2, stabilizing YBX1, upregulating SOX2, and enhancing CSC phenotype and Sunitinib resistance (Guo et al., 2022). In lung adenocarcinoma, YBX1 binds the MUC1 promoter, promoting CSC stemness and migration (Xie et al., 2021). In circRNA regulation, circRABL2B interacts with YBX1 to inhibit MUC5AC, suppressing CSC traits and enhancing drug sensitivity (Lu et al., 2023). YBX2 and hnRNPA2B1 are also linked to CSC traits. In endometrial cancer, high YBX2 correlates with increased stemness gene expression, higher ALDH1+ cells, enhanced serial sphere formation, and increased chemoresistance. YBX2 upregulates the cancer-testis antigen CT45, promoting CSC self-renewal and paclitaxel resistance, and is elevated in high-grade or advanced tumors (Suzuki et al., 2021). These studies reveal YBXs protein and m^5^C-dependent RNA nuclear export as critical mechanisms for CSC maintenance and therapy resistance, providing potential therapeutic strategies.

### 3.3 Functions and mechanisms of ac^4^C in CSCs

Multiple studies indicate that NAT10 and its catalyzed ac^4^C modification promote tumor progression in various cancers by maintaining CSC traits. In AML, NAT10 enhances translation of SLC1A4, HOXA9, and MENIN, promoting leukemia stem cell self-renewal and tumorigenesis (Zhang et al., 2024a). In colorectal and ovarian cancers, NAT10 stabilizes PGAM1 and CAPRIN1 mRNAs, increasing CSC sphere-forming ability, migration, invasion, and chemoresistance, thereby promoting tumor growth and metastasis (Zhang and Shen, 2025; Song and Cheng, 2025). In GBM, NAT10 mediates JARID2 mRNA ac^4^C modification, increasing protein levels, enhancing CSC stemness, and promoting malignant progression (Inoki et al., 2025). In NSCLC, NAT10 stabilizes CEBPA mRNA via ac^4^C modification, promoting CSC traits, proliferation, migration, and tumor growth (Qiu et al., 2026). Collectively, these findings demonstrate that ac^4^C RNA modification, primarily mediated by NAT10, plays a critical role in regulating CSC stemness, tumor progression, and therapy resistance by modulating the stability and translation of multiple oncogenic transcripts. These insights highlight the ac^4^C regulatory axis as a promising therapeutic target for suppressing CSC function across different malignancies.

### 3.4 Functions and mechanisms of m^1^A in CSCs

In HCC, m^1^A modification of tRNAs is significantly elevated, especially in liver CSCs, correlating with poor patient survival. The m^1^A methyltransferase complex TRMT6/TRMT61A is highly expressed in advanced HCC, increasing specific tRNA m^1^A modification, enhancing PPARδ translation, promoting cholesterol synthesis, activating Hedgehog signaling, maintaining liver CSC self-renewal, and driving tumorigenesis, inhibition of TRMT6/TRMT61A effectively suppresses liver cancer growth, highlighting it as a potential therapeutic target (Wang et al., 2021a). In contrast, the demethylase ALKBH3 does not affect HSC regeneration or maintenance, though its overexpression can correct differentiation bias in aged HSCs, indicating ALKBH3 mainly regulates aged stem cell function rather than CSC maintenance (He et al., 2020). Overall, m^1^A modification via TRMT6/TRMT61A drives liver CSC stemness and tumorigenesis, while ALKBH3 primarily regulates HSC differentiation.

### 3.5 Functions and mechanisms of m^7^G in CSCs

In cancers, m^7^G tRNA modification is catalyzed by METTL1, whose expression is often amplified and associated with poor prognosis. METTL1 deletion reduces m^7^G-modified tRNA abundance, disrupts the cell cycle, and suppresses tumorigenesis; conversely, METTL1 overexpression promotes cellular transformation and tumor formation (Orellana et al., 2021). Mechanistically, METTL1 increases m^7^G modification of specific tRNAs (e.g., Arg-TCT-4-1), remodeling the mRNA translatome, enhancing translation of cell cycle regulators and pro-growth proteins enriched in corresponding codons, thereby driving CSC-related proliferation and tumorigenesis. METTL1 and m^7^G tRNA modification represent potential targets for anticancer therapy (Orellana et al., 2021).

## 4 Cancer stem cells signaling pathways are regulated by RNA modifications

**FIGURE 1:**
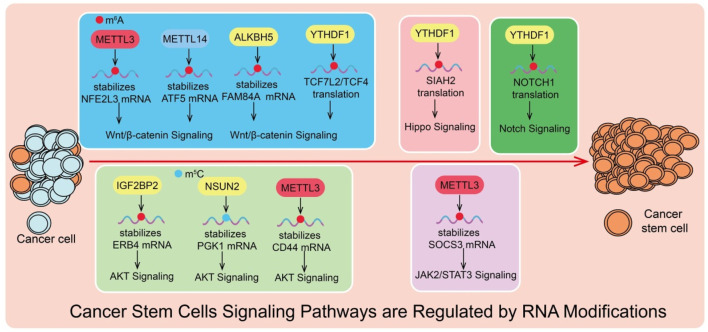
RNA modifications regulate signaling pathways that maintain cancer stem cells. m^6^A and m^5^C RNA modifications mediated by writers (e.g., METTL3, METTL14, NSUN2), erasers (ALKBH5), and readers (YTHDF1, IGF2BP2) modulate the stability or translation of target mRNAs. These modifications influence key signaling pathways—including Wnt/β-catenin, Hippo, Notch, AKT, and JAK2/STAT3, thereby promoting cancer stem cell maintenance and tumor progression.

As summarized in Figure 1, RNA chemical modifications play a critical role in regulating signaling pathways that sustain CSC properties. RNA modifications have emerged as important regulators of signaling pathways that govern CSC maintenance, self-renewal, and therapy resistance. By modulating mRNA stability, translation efficiency, and transcript localization, epitranscriptomic regulators dynamically fine-tune signaling networks that control CSC fate. Among these pathways, Wnt/β-catenin, Notch, PI3K–AKT–mTOR, JAK/STAT, and Hippo/YAP signaling have been extensively implicated in CSC biology, and growing evidence indicates that diverse RNA modifications participate in their regulation.

### 4.1 Wnt/β-catenin signaling are regulated by RNA modifications

The Wnt/β-catenin pathway is a core regulator of CSC self-renewal and stemness maintenance. Activation of Wnt signaling stabilizes β-catenin and promotes its nuclear translocation, where it induces transcription of stemness-associated genes such as c-MYC, SOX2, and OCT4, thereby maintaining CSC proliferation and an undifferentiated state. In addition, Wnt signaling contributes to metabolic reprogramming in CSCs by balancing glycolysis and oxidative phosphorylation to adapt to hypoxic or nutrient-limited tumor microenvironments (Liu et al., 2022). Increasing evidence suggests that RNA modifications function as an important regulatory layer that fine-tunes Wnt signaling activity. Among RNA modifications, m^6^A plays a prominent role in regulating the Wnt/β-catenin pathway. For example, in lung adenocarcinoma, OV6^+^ CSCs depend on METTL3, which stabilizes NFE2L3 mRNA through m^6^A modification. This process activates Wnt signaling, maintains CSC stemness, and promotes M2 macrophage infiltration, thereby establishing an immunosuppressive microenvironment. Targeting METTL3 effectively eliminates OV6^+^ CSCs, suppresses tumor progression, and enhances cisplatin sensitivity[ (Zhao et al., 2025b). Similarly, in colorectal and gastric cancers, METTL14 functions as a negative regulator of CSC stemness. Reduced METTL14 expression correlates with increased CSC traits and poor prognosis. Mechanistically, METTL14 promotes the m^6^A-dependent degradation of SCD1 and ATF5 transcripts, thereby suppressing Wnt/β-catenin or WDR74/β-catenin signaling activity (Xu et al., 2024). Interestingly, METTL14 expression itself can be epigenetically regulated; for example, histone H3 lactylation can increase METTL14 expression and influence CSC phenotypes (Zhang et al., 2025).

In addition to m^6^A writers, m^6^A readers and erasers also contribute to Wnt signaling regulation. The m^6^A demethylase ALKBH5 promotes colorectal CSC stemness by demethylating FAM84A mRNA, preventing β-catenin degradation and thereby enhancing Wnt signaling activity (Zhou et al., 2025b). Furthermore, the m^6^A reader YTHDF1 promotes translation of TCF7L2 (TCF4), a key transcriptional effector of Wnt signaling, thereby amplifying β-catenin activity and maintaining intestinal stem cell identity and tumorigenesis (Han et al., 2020). Beyond canonical m^6^A regulators, other RNA-binding proteins associated with RNA modification pathways may also influence Wnt signaling. For example, in intrahepatic cholangiocarcinoma, YBX1 enhances the expression of CSC markers including SOX9, OCT4, CD133, CD44, and EPCAM, thereby maintaining CSC stemness and reducing cisplatin sensitivity through activation of AKT/β-catenin signaling (Shi et al., 2024). Collectively, these studies demonstrate that RNA modifications—particularly m^6^A—regulate Wnt/β-catenin signaling at multiple levels, thereby controlling CSC stemness, tumor progression, and therapeutic resistance.

### 4.2 Notch signaling are regulated by RNA modifications

Notch signaling plays a key role in CSC differentiation decisions and fate maintenance (Artavanis-Tsakonas et al., 1999). Upon ligand binding, the Notch receptor is cleaved by γ-secretase, releasing the Notch intracellular domain (NICD), which translocates into the nucleus to activate downstream transcription factors such as HES and HEY, preventing premature differentiation and maintaining the CSC stem-like phenotype (Artavanis-Tsakonas et al., 1999). Notch also mediates CSC resistance to anti-tumor factors in the microenvironment, enhancing chemoresistance (Gallenstein et al., 2023). Emerging evidence indicates that RNA modifications can dynamically regulate the stability and translation of Notch pathway components. The m^6^A reader YTHDF1 has been reported to regulate Notch signaling in several cancers. In HCC, YTHDF1 binds m^6^A-modified NOTCH1 mRNA, enhancing its stability and translation, thereby promoting liver CSC self-renewal and resistance to multi-target tyrosine kinase inhibitors (Zhang et al., 2024b). Similarly, in colorectal cancer, YTHDF1-mediated recognition of m^6^A-modified NOTCH1 transcripts enhances CSC self-renewal, tumorigenesis, and resistance to chemotherapeutic agents such as oxaliplatin and 5-FU (Cheung et al., 2025). These findings suggest that m^6^A-mediated post-transcriptional regulation of Notch pathway components contributes to the maintenance of CSC plasticity and therapy resistance.

### 4.3 PI3K–AKT–mTOR signaling are regulated by RNA modifications

The PI3K–AKT–mTOR pathway is central to CSC metabolism, survival, and drug resistance (Glaviano et al., 2023). Activated PI3K phosphorylates AKT, which in turn activates mTORC1/2, promoting protein synthesis, glycolysis, and lipid metabolism to meet the high metabolic demands of CSCs and maintain stemness (Glaviano et al., 2023). Increasing evidence indicates that RNA modifications regulate key nodes within the PI3K–AKT–mTOR signaling network. Several m^6^A regulators influence CSC properties through the PI3K–AKT pathway. In ovarian cancer, IGF2BP2 interacts with circ_0000745, stabilizing the circRNA and maintaining CSC stemness and invasiveness through ERBB4-mediated activation of PI3K/AKT signaling (Wang et al., 2021b). In castration-resistant prostate cancer (CRPC), the lncRNA AGD1 promotes the stabilization of METTL13 via interaction with USP10, which in turn regulates CD44 mRNA stability through m^6^A modification, thereby activating the STAT3/PI3K–AKT pathway and promoting tumor stemness and docetaxel resistance (Wang et al., 2025b). Furthermore, in esophageal squamous cell carcinoma, the METTL14–miR-99a-5p–TRIB2 regulatory axis forms a positive feedback loop that enhances CSC properties and radioresistance. In this circuit, TRIB2 activates HDAC2 through the Akt/mTOR/S6K1 pathway, resulting in epigenetic repression of p21 and promoting tumor progression (Liu et al., 2021). Additionally, in AML, METTL3 promotes the translation of c-MYC, BCL2, and PTEN mRNAs via m^6^A modification, thereby maintaining leukemia cell proliferation and inhibiting differentiation (Vu et al., 2017). In addition to m^6^A, m^5^C modification also contributes to PI3K–AKT pathway activation. In gastric cancer, NSUN2 catalyzes m^5^C modification of PGK1 mRNA, which is recognized by the m^5^C reader YBX1, thereby enhancing PGK1 mRNA stability and activating the PI3K/AKT pathway to promote CSC stemness, proliferation, invasion, and glycolysis (Liu et al., 2025b). Overall, RNA modifications regulate the PI3K–AKT–mTOR signaling network through multiple mechanisms, thereby influencing CSC metabolism, survival, and therapeutic responses.

### 4.4 JAK/STAT signaling are regulated by RNA modifications

In CSCs, JAK/STAT signaling is primarily activated by cytokines such as IL-6 and IL-10, which trigger STAT3/STAT5 to drive transcription of stemness genes (SOX2, OCT4, NANOG), promoting self-renewal and proliferation (Hu et al., 2021). This pathway also enhances CSC resistance to apoptosis and adaptation to stress, supporting survival in inflammatory or therapeutic microenvironments. Among RNA modifications, m^6^A plays an important role in regulating JAK/STAT signaling in CSCs. In hepatocellular carcinoma, METTL3 is highly expressed in liver CSCs and correlates with tumor stage, grade, and lymph node metastasis. Mechanistically, METTL3 stabilizes SOCS3 mRNA through m^6^A modification, thereby activating the JAK2/STAT3 signaling pathway and promoting liver CSC self-renewal and tumorigenesis. Overexpression of SOCS3 suppresses CSC stemness, whereas activation of METTL3 enhances CSC phenotypes (Hu et al., 2024). These findings highlight the importance of m^6^A-mediated regulation of JAK/STAT signaling in maintaining CSC stemness and tumor progression.

### 4.5 Hippo/YAP signaling are regulated by RNA modifications

Hippo signaling, through nuclear YAP/TAZ transcription factors, regulates CSC proliferation, stemness, and migration (Kiang et al., 2024). Hyperactivation of YAP/TAZ signaling is frequently observed in solid tumor CSCs and contributes to enhanced self-renewal capacity, resistance to differentiation, and increased tumor invasiveness (Kiang et al., 2024). Recent studies suggest that m^6^A readers can influence Hippo signaling in CSCs. In triple-negative breast cancer (TNBC), the m^6^A reader YTHDF1 promotes the expression of SIAH2, which activates the Hippo pathway and enhances CSC stemness, proliferation, and chemoresistance (Wu et al., 2024). Together, these findings indicate that RNA modifications contribute to the regulation of Hippo/YAP signaling and play important roles in controlling CSC maintenance and tumor progression.

## 5 RNA modifications as novel CSC-Targeted therapeutic strategies and challenges

Recent studies have highlighted the critical role of RNA modifications in CSC stemness, self-renewal, and therapy resistance, positioning them as potential precision therapy targets. Targeting RNA modification enzymes can effectively suppress CSC functions, enhancing tumor sensitivity to conventional therapies. For example, inhibitors of METTL3 or demethylases (FTO, ALKBH5) regulate expression of CSC stemness genes, inhibit sphere formation, reduce stemness marker expression, and suppress tumor proliferation. Small molecule inhibitors targeting m^6^A readers, demethylases, and methyltransferases have made significant progress. YTHDC1 inhibitor YL-5092 blocks YTHDC1 binding to m^6^A substrates in AML, reducing mRNA stability, inducing leukemia cell apoptosis, and suppressing LSCs (Zhang et al., 2026). FTO inhibitors FB23/FB23-2 and ALKBH5 inhibitor DDO-2728 increase m^6^A levels by inhibiting demethylase activity, promoting AML cell differentiation and apoptosis (Huang et al., 2019). METTL3 inhibitor STM2457 reduces m^6^A modification of leukemogenic mRNAs, delaying AML progression and targeting LSCs (Yankova et al., 2021). YTHDF1 inhibitor tegaserod and IGF2BP2 inhibitor CWI1-2 block key m^6^A reader protein-mRNA interactions or downstream signaling, suppressing AML stem cell self-renewal and metabolism-related pathways (Weng et al., 2022). In addition, NAT10 ac^4^C methyltransferase inhibitor fludarabine suppresses AML stem cell self-renewal and tumor growth by inhibiting ac^4^C modification and metabolic reprogramming (Zhang et al., 2024a).

Collectively, these studies highlight the central role of RNA modification enzymes in CSC maintenance and leukemia progression, providing experimental and clinical rationale for developing selective small-molecule interventions. Combining RNA modification enzyme inhibitors with chemotherapy, radiotherapy, or immunotherapy may achieve synergistic effects and improve overall cancer treatment outcomes (Deng et al., 2023). However, CSC-targeted RNA modification strategies face challenges. Many RNA modification enzymes also function in normal stem cells; systemic inhibition may cause toxicity. RNA modification networks are complex, and inhibition of a single enzyme may be compensated by other pathways (Huang et al., 2020b). Optimal timing, dosage, and combination therapy strategies are required to avoid long-term effects on normal tissues. Despite these challenges, RNA modification enzymes remain promising CSC-targeted therapeutic strategies, potentially achieving efficient and safe intervention through precise targeting and combination therapy.

## 6 Conclusions and Perspectives

RNA modifications have been widely recognized as critical regulators of CSC functions, influencing mRNA stability, translation efficiency, nuclear-cytoplasmic transport, and signaling, thereby modulating CSC stemness, self-renewal, therapy resistance, and tumor relapse (Saw et al., 2024). Notably, individual RNA modifications do not act in isolation but form a complex multi-layered regulatory network, shaping CSC phenotypes via interactions and feedback mechanisms. This underscores their central role in tumor biology and suggests that targeting RNA modification enzymes may yield synergistic effects in combination therapy (Prasetyanti and Medema, 2017).

Future research, integrating advanced technologies, will achieve higher precision and spatial resolution. Single-cell epitranscriptomics (scEpitranscriptomics) may reveal CSC heterogeneity and differences in modification landscapes among subpopulations, elucidating dynamic mechanisms of cell-state transitions and stemness maintenance. Spatial transcriptomics can reveal CSC distribution within the tumor microenvironment and correlations between RNA modifications and local signals, guiding precise targeting. Dynamic monitoring of CSC state transitions combined with RNA modification data will enhance understanding of therapy resistance and relapse mechanisms. Furthermore, integrating RNA modification information with AI modeling can predict functional nodes within regulatory networks and inform personalized treatment strategies. In summary, RNA modifications represent a frontier in CSC research, deepening understanding of stemness mechanisms and providing abundant theoretical basis and potential targets for precision cancer therapy.

Despite emerging evidence highlighting the role of m^7^G RNA modification in CSC biology, current knowledge remains limited and largely centered on METTL1-mediated tRNA methylation (Wang et al., 2021a). Existing studies suggest that METTL1-dependent m^7^G modification can influence tRNA remodeling and translational control, thereby contributing to CSC maintenance and tumor progression. However, the mechanistic framework of m^7^G regulation in CSCs is still incomplete. Future studies should expand beyond METTL1 to investigate additional potential m^7^G writers, readers, or regulatory factors across diverse cancer types to better define the epitranscriptomic network governing CSC behavior. Moreover, it will be important to explore the functional significance of m^7^G modification in different CSC populations and tumor microenvironments, as CSCs from distinct cancers may exhibit unique regulatory dependencies. Another key direction is to elucidate the potential crosstalk between m^7^G modification and other epigenetic or epitranscriptomic marks, which may cooperatively regulate gene expression programs underlying CSC stemness and therapy resistance. Finally, rigorous validation of METTL1 as a therapeutic target—particularly through *in vivo* models and preclinical studies—will be essential to determine whether targeting the m^7^G regulatory axis can effectively suppress CSC-driven tumor initiation, progression, and relapse.
